# Forbidden Coherence Transfer of ^19^F Nuclei to Quantitatively Measure the Dynamics of a CF_3_-Containing Ligand in Receptor-Bound States

**DOI:** 10.3390/molecules22091492

**Published:** 2017-09-07

**Authors:** Yuji Tokunaga, Koh Takeuchi, Ichio Shimada

**Affiliations:** 1 Molecular Profiling Research Center for Drug Discovery, National Institute of Advanced Industrial Science and Technology (AIST), 2-3-26 Aomi, Koto-ku, Tokyo 135-0064, Japan; tokunaga.y@aist.go.jp (Y.T.); koh-takeuchi@aist.go.jp (K.T.); 2 Graduate School of Pharmaceutical Sciences, the University of Tokyo, 7-3-1 Hongo, Bunkyo-ku, Tokyo 113-0033, Japan

**Keywords:** NMR spectroscopy, dynamics, structural biology, drug development, trifluoromethyl

## Abstract

The dynamic property of a ligand in the receptor-bound state is an important metric to characterize the interactions in the ligand–receptor interface, and the development of an experimental strategy to quantify the amplitude of motions in the bound state is of importance to introduce the dynamic aspect into structure-guided drug development (SGDD). Fluorine modifications are frequently introduced at the hit-to-lead optimization stage to enhance the binding potency and other characteristics of a ligand. However, the effects of fluorine modifications are generally difficult to predict, owing to the pleiotropic nature of the interactions. In this study, we report an NMR-based approach to experimentally evaluate the local dynamics of trifluoromethyl (CF_3_)-containing ligands in the receptor-bound states. For this purpose, the forbidden coherence transfer (FCT) analysis, which has been used to study the dynamics of methyl moieties in proteins, was extended to the ^19^F nuclei of CF_3_-containing ligands. By applying this CF_3_–FCT analysis to a model interaction system consisting of a ligand, AST-487, and a receptor, p38α, we successfully quantified the amplitude of the CF_3_ dynamics in the p38α-bound state. The strategy would bring the CF_3_-containing ligands within the scope of dynamic SGDD to improve the affinity and specificity for the drug-target receptors.

## 1. Introduction

The affinity and specificity of small molecules to their targets need to be improved in the hit-to-lead and the lead optimization processes in drug development. Target specificity is known to be associated with the thermodynamic properties of binding [1]. Ligands with larger enthalpic contributions to the receptor-binding free energy are expected to show better target specificity and to be less susceptible to drug-resistance mutations [2,3,4]. Such enthalpy-driven bindings are usually derived from multiple site-specific intermolecular interactions, such as hydrogen bonds and van der Waals interactions. The static structures of ligand–receptor complexes, mainly provided by X-ray crystallography, are able to identify those intermolecular interactions. However, the energetic contribution of each interaction cannot be readily predicted from a static structure. In this regard, the mobility of atoms in and near the intermolecular interaction site is expected to reflect the rigidity and strength of the local interaction [5,6,7]. Therefore, the current structure-guided drug development (SGDD) approaches, which rely largely on static structures, would be improved by taking the dynamic aspects of interactions into account and by developing experimental strategies to quantify the amplitude of motions.

Solution nuclear magnetic resonance (NMR) techniques have greatly contributed to pharmaceutical development at various stages [8,9,10]. NMR plays a critical role in the supply of intermolecular distance information about ligand–receptor complexes, especially for the cases where solving the crystal structures of complexes is difficult. More importantly, the solution NMR techniques also provide information about the dynamics of both the ligands and the target biomolecules at an atomic resolution, with timescales ranging from picosecond to hour [11,12,13,14,15,16]. Among them, the forbidden coherence transfer (FCT) analyses have been established to quantify the amplitude of the ps–ns timescale motion of methyl (CH_3_) groups in macromolecules [17]. The CH_3_–FCT analyses extract two relaxation parameters: the intramethyl ^1^H–^1^H dipolar cross-correlated relaxation rate, *η*, and the sum of the dipolar cross-relaxation rates with external protons, *δ*. The parameter *η* is directly proportional to the generalized order parameter, *S*_axis_^2^, which reports the amplitude of motion of the methyl threefold axis. The parameter *δ* is sensitive to the presence of remote protons within 3 Å, thus providing information about the surface complementarity if used in a ligand–receptor complex [18]. The advantages of the CH_3_–FCT approach in ligand optimization were exemplified in our preceding research [18]. Here, the CH_3_–FCT analyses were applied to a μM-affinity peptide ligand that binds to a drug-target kinase, to identify the methyl-bearing residues with a large motional amplitude and low surface complementarity in the kinase-bound state. The substitution of the residue with such propensities by a bulkier amino acid substantially improved the affinity and thermodynamic properties of the ligand.

Here, we report the extension of the FCT analysis to trifluoromethyl (CF_3_) groups. Approximately 25% of therapeutic drugs on the market contain at least one fluorine atom, and the CF_3_ group is one of the major fluorine-containing substituents [19]. Since recent advances in medicinal chemistry allow easy incorporation of fluorine atoms to specific sites of compounds [20,21], fluorine is frequently introduced at the lead optimization stage to enhance binding potency, oral availability, and/or metabolic stability [22,23]. The enhancement of the binding potency can be primarily attributed to the fluorine-mediated intermolecular interactions, including hydrogen bonds, halogen bonds, and van der Waals interactions. However, the strong electron-withdrawing propensity of fluorine also affects the interactions of remote functional groups, and the bulky fluorine substituent often modulates the dynamics of ligands in both the free and bound states. Such a pleiotropic nature makes the effects of fluorine incorporation difficult to predict. Therefore, implementation of a novel experimental approach to characterize the interaction and dynamics of compounds at fluorinated sites is of importance. As ^19^F is the 100% naturally abundant ½-spin nucleus with the next largest gyromagnetic ratio to the proton, it is feasible for use in NMR applications, and affords high sensitivity. In addition, the analysis of ^19^F-NMR spectra is straightforward, owing to the large chemical shift distribution (~100 ppm) and no background signals. These features make ^19^F-NMR an attractive approach in drug discovery [24], and the CF_3_ moiety has been extensively used in drug-screening procedures due to its high sensitivity and relatively narrow line width [25,26].

In this report, we developed the CF_3_–FCT analysis to quantitatively measure the dynamics of a CF_3_-containing ligand in the receptor-bound state. The characteristics of the CF_3_ groups that are distinct from those of the CH_3_ groups—including the smaller gyromagnetic ratio, the longer spin–spin and van der Waals contact distances and the shorter ^19^F transverse relaxation times—were considered. We applied the strategy to a ligand, AST-487, complexed with a drug-target protein kinase, p38α, and demonstrated that the *η* relaxation parameter is readily obtained to quantitatively measure the amplitude of the dynamics of the CF_3_ moiety. In addition, the comparison of the FCT profile of the ligand in the non-deuterated receptor-bound state, relative to that of the perdeuterated receptor-bound state, indicated that the strategy would provide the surface complementarity information at the CF_3_ site.

## 2. Results and Discussion

In the FCT analyses, the time (*T*)-dependent buildup of the intensity ratio of the multiple quantum coherence (*I_MQC_*) over the single quantum coherence (*I_SQC_*) is used to determine the motion- and distance-associated relaxation parameters *η* and *δ*, respectively, as following,

(1) $$\frac{I_{MQC}}{I_{SQC}}=\frac{A\eta \cdot tanh\left(\sqrt{\eta^{2}+\delta^{2}}T\right)}{\sqrt{\eta^{2}+\delta^{2}}-\delta \cdot tanh\left(\sqrt{\eta^{2}+\delta^{2}}T\right)}$$

where *A* is 0.5 and 0.75 for double and triple quantum coherence, respectively. Since the tetrahedral geometry and the rapid rotation around the threefold axis are common, the FCT analyses of both the CH_3_ and CF_3_ groups can be described by the same equation. Analogous to the FCT analyses for CH_3_ groups [17], the equations defining the intra-trifluoromethyl ^19^F–^19^F dipolar cross-correlated relaxation rate $\eta_{F}$ and the heteronuclear dipolar cross relaxation of ^19^F to external nuclei $\delta_{F}$ are described as below,

(2) $$\eta_{F}=\frac{R_{2,\;F}^{fast}-R_{2,\;F}^{slow}}{2}\approx \frac{9}{10}{\left[P_{2}\left(cos\theta_{axis,FF}\right)\right]}^{2}\frac{S_{axis}^{2}\gamma_{F}^{4}{ћ}^{2}\tau_{C}}{r_{FF}^{6}}$$

(3) $$\delta_{F}=-4\underset{ext}{\sum }\left(\frac{1}{20}\right)\frac{{ћ}^{2}\gamma_{F}^{2}\gamma_{X}^{2}\tau_{C}}{r_{FXext}^{6}}$$

where $R_{2,\;F}^{fast}$ and $R_{2,\;F}^{slow}$ are relaxation rates of fast and slow ^19^F single quantum transitions, respectively. $P_{2}\left(x\right)=(1/2)\left(3x^{2}-1\right)$, $\theta_{axis,FF}$ is the angle between the trifluoromethyl threefold axis and the vector connecting two fluorine nuclei. $S_{axis}^{2}$ is the generalized order parameter, $\gamma_{F}$ is the gyromagnetic ratio of F spins, $\gamma_{X}$ is the gyromagnetic ratio of remote F or H spins, ћ is the reduced Planck constant, $\tau_{C}$ is the rotational correlation time of the molecule, $r_{FF}$ is the distance between two fluorine nuclei in a trifluoromethyl group, and $r_{FXext}$ is the averaged distance between three fluorine atoms and an external H or F spin. Similar to that in the FCT analysis of a CH_3_ group, the slope of the *I_MQC_*/*I_SQC_* buildup curve depends mostly on the $\eta_{F}$ values. Therefore, the local dynamics of the ligand methyl groups in the ligand–receptor complex can be obtained from the initial CF_3_–FCT buildup. Analogously, the *δ*_F_ values, which reflect the vicinity of the CF_3_ groups with external protons, i.e., surface complementarity, would be reflected in the plateau value of the *I_MQC_*/*I_SQC_* buildup.

However, some differences should be considered for assessing the practical applicability of the FCT analysis to the CF_3_ groups. The smaller gyromagnetic ratio and the longer spin–spin distances in the CF_3_ group, as compared to those in the CH_3_ group, lead to smaller *η* and *δ* values in the CF_3_–FCT analyses. The gyromagnetic ratios for ^19^F and ^1^H are 251.662 × 10^6^ rad/s/T and 267.513 × 10^6^ rad/s/T, respectively. Interatomic ^19^F–^19^F and ^1^H–^1^H distances for the CF_3_ and CH_3_ groups are 2.164 Å and 1.813 Å, respectively. Assuming the same $S_{axis}^{2}$ and $\tau_{C}$ values, these differences make the *η*_F_ value 0.27-times smaller as compared to the *η*_H_ value. Therefore, the slope of the CF_3_–FCT buildup would be less steep, as compared to that of the CH_3_–FCT. This also means that the FCT profile of CF_3_ requires a much longer mixing time to reach the plateau value, and thus, is less sensitive to the presence of external protons at practical mixing times, which are defined by the ^19^F transverse relaxation times. In addition, the δ value itself tends to be smaller, as the van der Waals contact distance of fluorine is longer for a ^19^F–^1^H pair (2.55 Å) as compared to that for a ^1^H–^1^H pair (2.4 Å). At the van der Waals contact distance, the *δ* value is 0.61 times smaller for ^19^F as compared to that for ^1^H.

**Figure 1:**
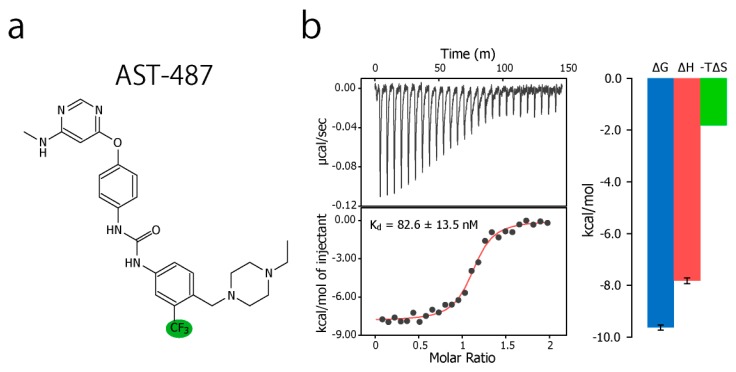
Chemical structure of AST-487 and its binding thermodynamics against p38α. (**a**) The chemical structure of AST-487, with the CF_3_ moiety highlighted in green; (**b**) The isothermal titration calorimetry (ITC) profile and thermodynamic properties of the interaction between AST-487 and p38α. In the ITC experiment, 50 μM AST-487 was titrated to 5 μM p38α at 25 °C.

**Figure 2:**
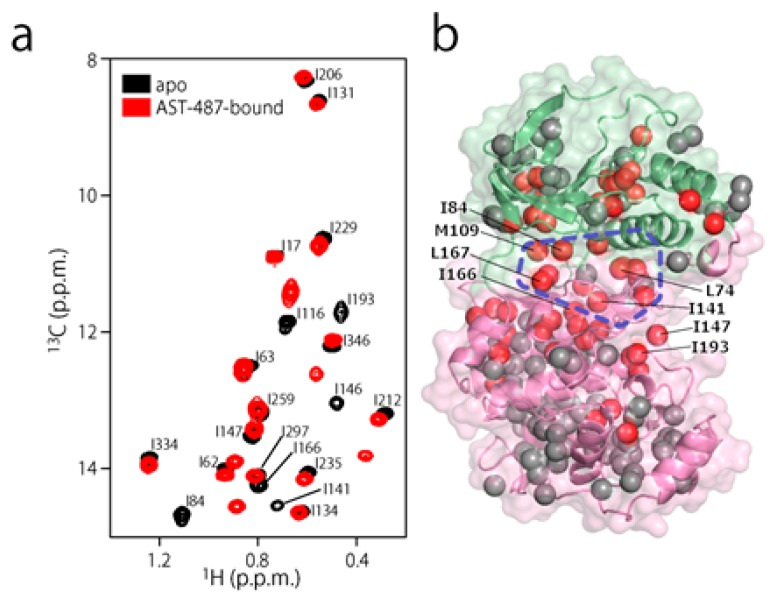

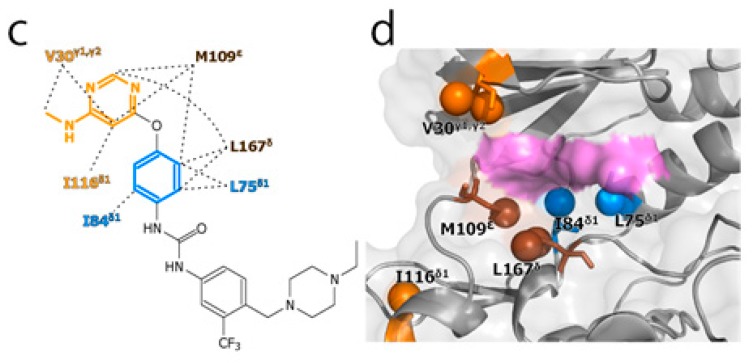
Structural characterization of the interaction between AST-487 and p38α. (**a**) The ^1^H–^13^C band-selective optimized-flip-angle short-transient–heteronuclear multiple quantum coherence (SOFAST–HMQC) spectra (Ile-*δ*1 region) of p38α with ^1^H/^13^C isotope labeling at the methyl positions of isoleucine (*δ*1), leucine, valine, and methionine in a uniformly deuterated background ([ILVM–methyl–^1^H/^13^C, U-^2^H] p38α) in the presence (red) and absence (black) of an equimolar concentration of AST-487; (**b**) The structural mapping of the chemical shift perturbations (CSPs) induced by the addition of AST-487. Methyl groups with CSPs larger than the linewidth are shown as red spheres in the structure of p38α in the apo state (Protein Data Bank (PDB) code: 1P38) [39]. The ATP-binding site is indicated by the blue broken line; (**c**) The intermolecular nuclear Overhauser effects (NOEs) observed between AST-487 and p38α are illustrated by broken lines. For Leu-167, only one resonance of the two *δ*-methyl groups was identified with no stereospecific assignments; thus, it is labeled as “*δ*”. The pyrimidine ring and the methylamino substituent are colored orange, while the ether-linked phenyl ring is colored blue; (**d**) The methyl sites with intermolecular NOEs with AST-487 are shown as spheres in the p38α structure (PDB code: 1A9U) [40]: orange methyl sites represent NOEs with the pyrimidine ring or its methylamino substituent; blue methyl sites represent NOEs with the ether-linked phenyl ring; and brown methyl sites represent NOEs to both. The surface of a representative p38α inhibitor, SB203580, is colored purple to indicate the typical compound-binding site.

To experimentally evaluate the CF_3_–FCT analysis, we used p38α, a 42 kDa mitogen-activated protein kinase, as a receptor. p38α is a major drug target for inflammatory diseases such as rheumatoid arthritis and Crohn’s disease [27,28]. For the ligand, we selected AST-487, which reportedly showed the highest p38α-binding or inhibition potency among CF_3_-containing compounds in the ChEMBL Kinase SARfari database (Figure 1a) [29]. AST-487 was originally identified as a receptor tyrosine (RET) kinase inhibitor [30]. However, it is shown to bind also to p38α with a dissociation constant (*K*_d_) of 72 nM [31]. We verified the AST-487 binding to p38α by an isothermal titration calorimetry (ITC) experiment, and observed a 1:1 exothermic interaction with a *K*_d_ value of 82.6 ± 13.5 nM (Figure 1b), which was identical to the reported value within the error. To further confirm that AST-487 binds to a specific site of p38α, we carried out a chemical shift perturbation (CSP) experiment, in which AST-487 was titrated to p38α with ^1^H/^13^C isotope labeling at the methyl positions of isoleucine (*δ*1), leucine, valine, and methionine in a uniformly deuterated background ([ILVM–methyl–^1^H/^13^C, U-^2^H] p38α) (Figure 2a and Supplementary Figure S1). Reflecting the high affinity of the complex, the ^1^H–^13^C resonances of p38α were perturbed in a slow exchange manner, saturating at one molar equivalent (Supplementary Figure S1). Methyl sites with substantial CSPs were mainly distributed in and near the ATP-binding site (Figure 2b), which is consistent with the canonical interaction mode of ATP-competitive inhibitors. In fact, intermolecular nuclear Overhauser effects (NOEs) were observed between the protons of AST-487 and the methyl protons in the ATP-binding site of p38α (Figure 2c,d). Extensive NOEs were observed for the pyrimidine group and ether-linked ring of AST-487 (Figure 2c). In particular, NOEs were observed for the pyrimidine group and the p38α methyl protons deeper in the ATP-binding cleft (orange spheres in Figure 2d). This NOE pattern is consistent with the binding mode of the AST-487–mitogen-activated protein kinase kinase 4 (MEK4) complex, which was previously analyzed by computational docking and saturation transfer difference (STD) NMR experiments, where the pyrimidine group of AST-487 resides deep in the binding cleft [32]. In contrast, we detected no intermolecular NOEs for the trifluoromethyl-phenyl ring and 4-ethyl-piperazin groups. This might be due to the lack of protons around these groups in the p38α bound state, or to the less-efficient NOE transfer caused by fast local dynamics. Such an ambiguity can be clarified by quantifying the amplitude of the local dynamics. In addition, as described above, AST-487 binds a broad range of kinases including Tyr kinases (e.g., RET kinase) and Ser/Thr kinases (e.g., MEK4 and p38α). Therefore, the dynamics information would contribute to the rational design of modifications for achieving better binding potency and the desired target specificity.

**Figure 3:**
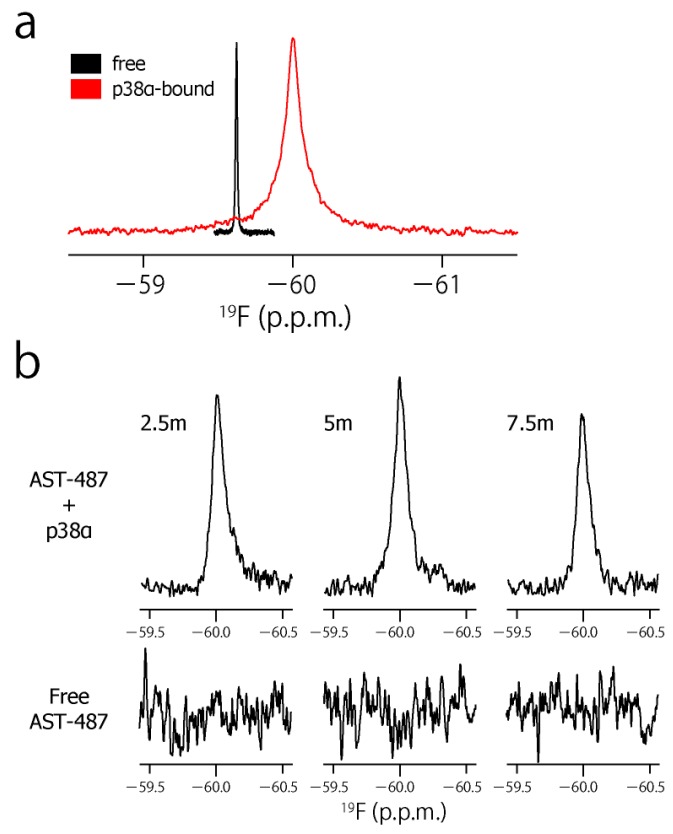

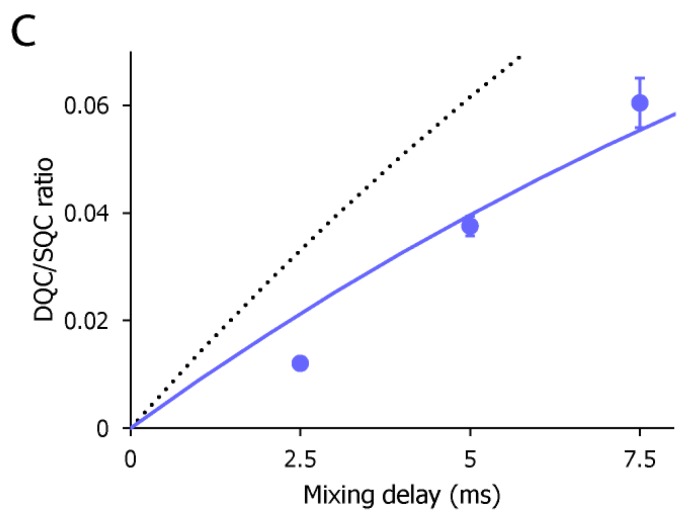
The CF_3_–FCT data of AST-487 complexed with p38α. (**a**) Overlay of the ^19^F–1D spectra of AST-487 in the presence (red) and absence (black) of perdeuterated p38α; (**b**) Spectral regions of the CF_3_ resonance of AST-487 bound to perdeuterated p38α (upper) and those in the absence of p38α (lower) in the ^19^F–DQC (double quantum coherence) spectra at mixing times of 2.5, 5 and 7.5 ms; (**c**) The time-dependent evolution of the *I*_DQC_/*I*_SQC_ ratio. Error bars are estimated from the signal-to-noise ratios. The fitting curve is drawn as a solid line. For comparison, a simulated curve with $S_{axis}^{2}$ = 1 (*δ* = −29 s^−1^) is shown as a dotted line. SQC: single quantum coherence.

To characterize the dynamic property of the CF_3_ group of AST-487 in the p38α-bound state, we observed the ^19^F resonance of the CF_3_ moiety of AST-487 in the presence of p38α. The ^19^F resonance in the presence of p38α was observed at −59.97 ppm, which was high field-shifted by 0.32 ppm from −59.65 ppm in the free state (Figure 3a). This resonance is derived from AST-487 in the bound state, as the binding of AST-487 to p38α occurs in the slow exchange manner and saturates at one molar equivalent (Supplementary Figure S1). Then, we performed the double quantum coherence (DQC) and single quantum coherence (SQC) measurements of AST-487 complexed with perdeuterated p38α. In these experiments, a broadband ^1^H decoupling was applied throughout the pulse sequence to suppress the ^1^H–^19^F scalar couplings. Mixing delays were set to 2.5, 5 and 7.5 ms, taking the signal decay due to the ^19^F transverse relaxation into consideration (Supplementary Figure S2). Although no signal was observed in the DQC spectra of the free AST-487, the buildup of a DQC signal was clearly observed in a mixing time-dependent manner for AST-487 in the p38α-bound state (Figure 3b). This supports that the signal observed in the p38α-bound state is derived from the FCT phenomenon, which is allowed only under high-molecular-weight conditions. Thus, the results confirmed that the FCT measurement is applicable to the CF_3_ group.

To estimate the motional amplitude of the CF_3_ moiety, the *I_DQC_*/*I_SQC_* buildup was fitted to Equation 1 (Figure 3c). Here, we assumed *δ_F_* = −28 s^−1^, taking account of the intramolecular dipolar cross-relaxation from two adjacent protons at the van der Waals distance. The dipolar contribution from perdeuterated p38α would be negligible. Under this assumption, the *η_F_* value was determined as 18.3 ± 1.96 s^−1^. This corresponds to the Lipari–Szabo model-free $S_{axis}^{2}$ of 0.64 ± 0.069. The estimated order parameter indicates that the CF_3_ group and its directly attached phenyl ring of AST-487 retain a substantial mobility in the p38α-bound state. This complements the lack of the NOE-based information about the intermolecular interaction for the trifluoromethyl-phenyl group.

We further investigated the effect of intermolecular dipole interactions on the CF_3_–FCT profile. To this end, we used non-deuterated p38α, instead of perdeuterated p38α, to introduce intermolecular dipole–dipole interactions between the CF_3_ group of AST-487 and the protons of p38α. The linewidth of the AST-487 ^19^F resonance complexed with non-deuterated p38α was broader than that with perdeuterated p38α (102 and 72 Hz, respectively; Supplementary Figure S3a), indicating a considerable increase in dipole–dipole interactions. The *I_DQC_*/*I_SQC_* ratio of the CF_3_ moiety at a mixing time of 5 ms was 24% smaller in the non-deuterated p38α-bound state than that in the perdeuterated p38α-bound state (Supplementary Figure S3b). This could be ascribed to the enhancement of *δ_F_* by the p38α protons, indicating that the CF_3_ group of ATS-487 is at least partially buried in the binding pocket of p38α. Thus, the result demonstrated that the surface complementarity information could be extracted from the CF_3_–FCT analysis.

In summary, we demonstrated that the CF_3_–FCT analyses are capable of characterizing the fast timescale motion and surface complementarity of the CF_3_ group of a small ligand in the receptor-bound state. The extension of the FCT analyses to the CF_3_ group would contribute to expanding the applicability of the dynamic SGDD to CF_3_-containing molecules. The analyses provide important ways to proceed with the hit-to-lead and lead optimization processes to obtain a small compound with improved affinity and specificity for the drug-target biomolecules. Nevertheless, the utility of the CF_3_–FCT analyses would not be limited to the field of drug development: the strategy might also be useful to characterize the functional dynamics of proteins. CF_3_ groups have been chemically introduced to monitor the conformational equilibrium of proteins in structural analyses that are difficult to tackle by other methods [33,34,35,36,37,38].

## 3. Materials and Methods

The compound AST-487 (molecular weight 529.6 Da, Purity 99.90%, Catalog No. SYN1210) was purchased from AK Scientific (Union City, CA, USA) and used without further purification. Powder of AST-487 was dissolved in DMSO-(*d*_6_) at a concentration of 100 mM, and small aliquots were stored at −30 °C.

### 3.1. Preparation of the Recombinant p38α

Overexpression and purification of the recombinant p38α were performed following the procedure as reported previously [41]. Briefly, the *E. coli* strain BL21 (DE3) was transformed with the pET15b plasmid harboring an *N*-terminally hexahistidine-tagged human full-length p38α sequence between the NcoI and XhoI restriction sites of the plasmid. Bacterial colonies grown on freshly prepared LB agar plates, containing 100 μg/mL ampicillin, were inoculated into a 5 mL LB medium and cultured at 37 °C overnight. For the production of perdeuterated p38α, the overnight culture was mildly centrifuged and the resultant cell pellet was gently resuspended in a 100 × volume of D_2_O-based M9 medium containing ^15^NH_4_Cl and ^2^H_7_-d-glucose as the sole nitrogen and carbon sources, respectively. When an optical density at 600 nm (OD_600_) of 0.6 was reached, the culture temperature was lowered to 16 °C. After 30 min, protein overexpression was induced by the addition of 0.6 mM isopropyl-β-d-thiogalactopyranoside (IPTG). For preparing [U–^2^H/^15^N, ILVM–methyl–^1^H/^13^C] p38α sample used in the p38α-observed NMR experiment, methionine and precursors of isoleucine, leucine and valine were supplemented 30 min before induction at the following concentrations: 50 mg/L [ε-^13^C] l-methionine; 90 mg/L [3-methyl-13C, 3,4,4,4-D4] α-ketoisovalerate, sodium salt; and 70 mg/L [methyl-^13^C,3,3-D_2_] α-ketobutyrate, sodium salt [42]. H_2_O-based M9 medium with no isotope enrichment was used for the preparation of non-deuterated p38α sample. Bacterial cells were collected by centrifugation and the cell pellet was stored at −80 °C until purification. The cell suspension was sonicated on ice to lyse cells. The supernatant was subjected to metal–chelate affinity chromatography by using a His60 Ni Superflow column (Takara Bio, Shiga, Japan), followed by size-exclusion chromatography using a HiLoad 16/60 Superdex 200 prep grade column (GE Healthcare, Little Chalfont, UK). The elution fraction was concentrated in the buffered solution containing 25 mM Tris-HCl (pH 7.5), 150 mM NaCl and 5 mM dithiothreitol (DTT), by ultrafiltration using an Amicon Ultra centrifugal device (MWCO 10 kDa, 4 mL, Merck Millipore, Darmstadt, Germany). The concentration of p38α was determined by measuring the ultraviolet absorbance at a wavelength of 280 nm and using an extinction coefficient of 47,850 L/mol/cm.

### 3.2. NMR Sample Preparation

All NMR samples were prepared in the buffered solution containing 25 mM Tris(D_11_)-DCl (pH 7.7), 150 mM NaCl and 10 mM DTT-(D_10_) in 95/5 D_2_O/DMSO-(*d*_6_). An aliquot of the p38α stock in the H_2_O-based buffer solution was buffer-exchanged to the DMSO-free NMR buffer by ultrafiltration using an Amicon Ultra device (MWCO 3 kDa, 0.5 mL). After adjusting the volume, the AST-487 solution and DMSO-(*d*_6_) were added to have the final concentrations of 1 mM AST-487 and 5% (*v*/*v*) DMSO-(*d*_6_). The concentrations of p38α were 150 μM and 400 μM for p38α-observed ^1^H–^13^C correlation experiments and AST-487-observed ^19^F-detected experiments, respectively. To the latter samples, trifluoroacetic acid (TFA) was supplemented at a concentration of 500 μM for chemical shift calibration.

### 3.3. NMR Experiments

Two dimensional ^1^H–^13^C SOFAST–HMQC [43,44] spectra of p38α in the absence and presence of AST-487 were acquired on a Bruker Avance 600 spectrometer equipped with a TXI cryoprobe operating at 298 K. The numbers of data points for the direct ^1^H and the indirect ^13^C dimensions were 1024 and 512, with maximum acquisition times of 61.1 and 74.8 ms, respectively. Twelve transients were acquired per free induction decay (FID), with an interscan delay of 0.3 s. Time domain data were processed with TopSpin2.1 software (Bruker BioSpin, Billerica, MA, USA) and analyzed by Sparky [45]. Proton and carbon chemical shifts were calibrated using 4,4-dimethyl-4-silapentane-1-sulfonic acid (DSS) as the external standard [46]. Methyl sites experiencing CSPs larger than the linewidth upon AST-487 binding were classified as perturbed.

The rotational correlation time (*τ*_c_) of p38α was determined as 24 ns in 85/10/5 H_2_O/D_2_O/DMSO-based solution by transverse relaxation-optimized spectroscopy for rotational correlation times (TRACT) experiments [47]. Considering the increased viscosity due to higher D_2_O content, the *τ*_c_ of p38α in 95/5 D_2_O/DMSO-based solutions used for FCT experiments was estimated to be 29 ns.

One-dimensional ^19^F–1D and the CF_3_–FCT experiments were performed on a Bruker Avance 600 (Bruker BioSpin, Billerica, MA, USA) spectrometer equipped with a QCI-F cryoprobe at 298 K. The pulse sequence for the one-dimensional ^19^F–DQC and SQC experiments were analogous to those for proton DQC and SQC measurements, respectively [48]. The DQC and SQC experiments were performed with complex data points of 8096 and an acquisition time of 151 ms. For the DQC and SQC measurements, 46,080 and 1024 transients, respectively, were acquired with a recycling delay of 1.5 s. The mixing times were 2.5, 5.0 and 7.5 ms for experiments using perdeuterated p38α, and 5 ms for those using non-deuterated p38α. The ^19^F carrier frequency was set at −58.84 ppm. The WALTZ-16 composite pulse decoupling [49] was applied to proton spins from the excitation to the end of the acquisition period to suppress any potential ^1^H–^19^F scalar couplings. The time domain data were processed and analyzed on the TopSpin3.1 software (Bruker Biospin), and the chemical shifts of ^19^F resonances were calibrated by using TFA as the internal standard.

### 3.4. ITC Experiments

The MicroCal VP-ITC calorimeter (Malvern, Malvern, UK) was used to determine the affinity and thermodynamic parameters associated with the binding of AST-487 to p38α. AST-487 and p38α were dissolved in the buffered solution (ITC buffer), containing 50 mM (*N*-2-hydroxyethylpiperazine-*N*-2-ethane sulfate (HEPES)–NaOH (pH 7.5), 150 mM NaCl, 1 mM tris(2-carboxyethyl)phosphine hydrochloride (TCEP), 4% (*v*/*v*) glycerol and 5% (*v*/*v*) DMSO. The ITC buffer was freshly prepared before use, passed through a 0.22-μm membrane filter and degassed by sonication. The 100 mM stock of AST-487 was diluted 1:1000 with the ITC buffer and centrifuged. The resultant supernatant was filtered through a 0.22-μm syringe filter. A stock solution of p38α was exchanged to the ITC buffer by ultrafiltration using an Amicon Ultra (Merck Millipore, Darmstadt, Germany) centrifugal device (MWCO 10 kDa) and filtered through a 0.22-μm syringe filter. A total of 27 aliquots of 50 μM AST-487 in the titration syringe (2 μL at the initial titration point and 10 μL at successive points) were added to the cell filled with 5 μM p38α, with an interval of 350 s between each pair of titration points at 25 °C. The reference titration data was acquired with the same titration procedure, except that the ITC buffer was used instead of the p38α solution. After subtracting the reference data and omitting the initial titration point, the molar heat–ligand concentration profile was fitted to the one-to-one binding model by using the Origin software (OriginLab Corporation).

## Supplementary Materials

Supplementary materials are available online. Figure S1: ATS-487 concentration-dependent spectral changes of p38α, Figure S2: Estimation of the applicable mixing delays for CF_3_–FCT analyses of the ATS-487-p38α complex, and Figure S3: The effect of receptor protonation on the ^19^F linewidth and the FCT build up.
