# Supplementary material for: Forbidden Coherence Transfer of 19F Nuclei to Quantitatively Measure the Dynamics of a CF3-Containing Ligand in Receptor-Bound States
Source: Molecules. 2017 Sep 7;22(9):1492. doi: 10.3390/molecules22091492 (PMC6151541; doi:10.3390/molecules22091492)
Supplement: Supplementary file 1 [file molecules-22-01492-s001.pdf]

Electronic Supplementary Information

Forbidden coherence transfer of ^{19}F nuclei to quantitatively measure the dynamics of a CF_3-containing ligand in receptor-bound states

Yuji Tokunaga [1], Koh Takeuchi [1] and Ichio Shimada [1,2,*]

[1] Molecular Profiling Research Center for Drug Discovery, National Institute of Advanced Industrial Science and Technology (AIST), 2-3-26 Aomi, Koto-ku, Tokyo 135-0064, Japan.; tokunaga.y@aist.go.jp, koh-takeuchi@aist.go.jp

[2] Graduate School of Pharmaceutical Sciences, the University of Tokyo, 7-3-1 Hongo, Bunkyo-ku, Tokyo 113-0033, Japan.; shimada@iw-nmr.f.u-tokyo.ac.jp

* Correspondence: shimada@iw-nmr.f.u-tokyo.ac.jp; Tel.: +81-3-3815-6540

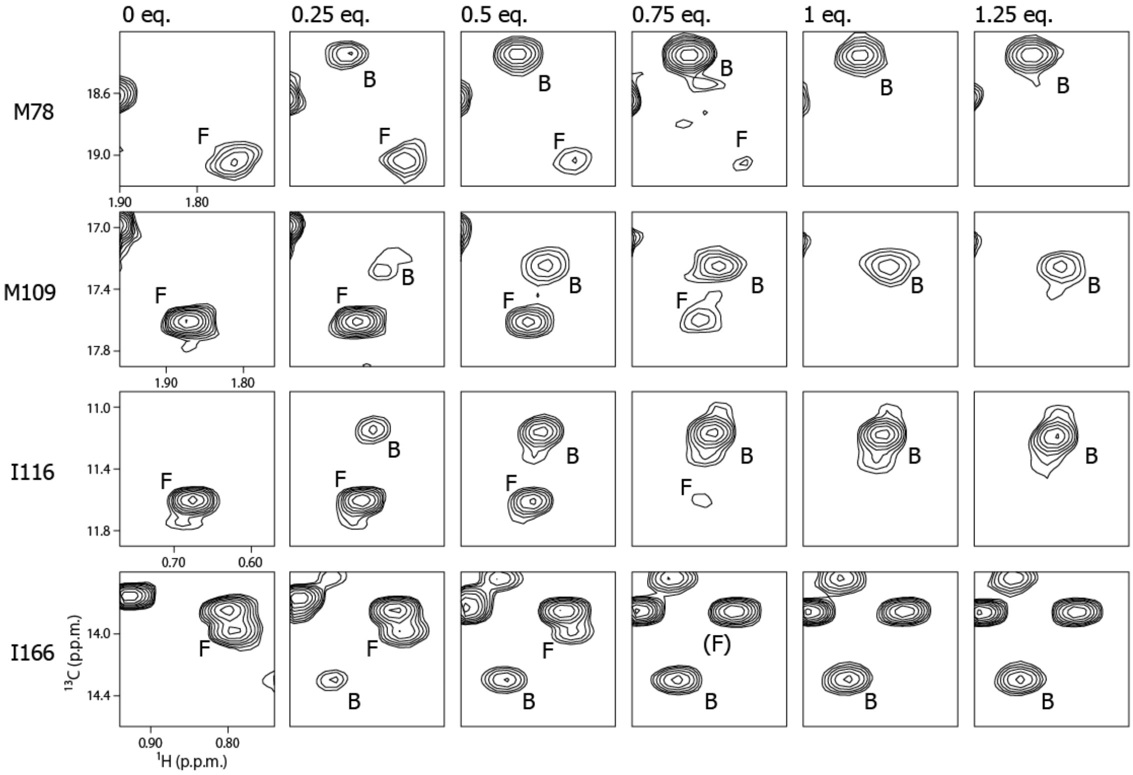

Supplementary Figure S1. ATS-487 concentration-dependent spectral changes of p38α. Regions of ^{1}H-^{13}C HMQC spectra of 50 μM [ILVM-methyl-^{1}H-^{13}C] p38α including Met-78, Met-109, Ile-116, and Ile-166 are shown from top to bottom. AST-487 was supplemented at 0.25, 0.5, 0.75, 1, and 1.25 molar equivalents of p38α (left to right). Resonances derived from the free and AST-487-bound states are labeled with "F" and "B", respectively. The resonance of Ile-116 in the free state at the 0.75 eq. AST-487 concentration is below the contour level of the figure; thus, it is labeled in parentheses.

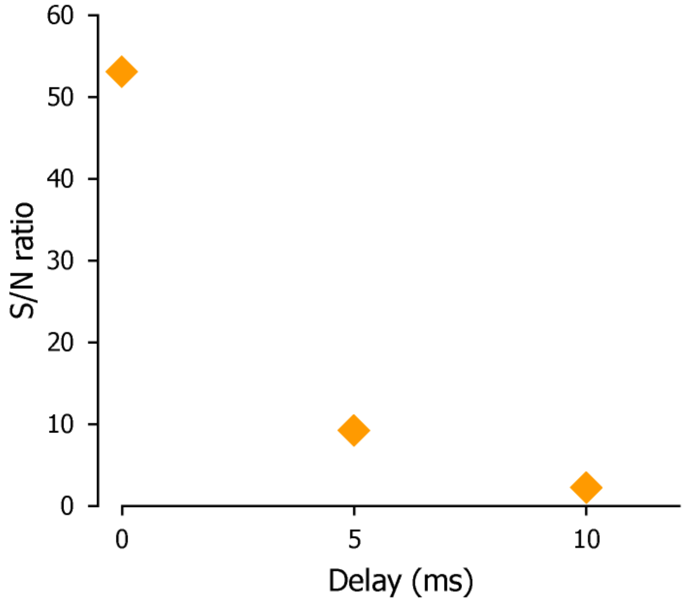

Figure S2

Supplementary Figure S2. Estimation of the applicable mixing delays for CF_3-FCT analyses of the ATS-487-p38α complex. Signal-to-noise ratios of the ^{19}F SQC resonance of 400 μM AST-487 complexed with p38α were measured at mixing delays of 0.004, 5, and 10 ms. The number of transients is 32. At the mixing delay of 10 ms, the S/N ratio decayed below 5% of that of 0.004 ms. Therefore, it was suggested that a mixing delay shorter than 10 ms would be realistic in this case. While the experiment was carried out with a 600 MHz spectrometer, the smaller magnetic field can be used to minimize the relaxation enhancement due to chemical shift anisotropy.

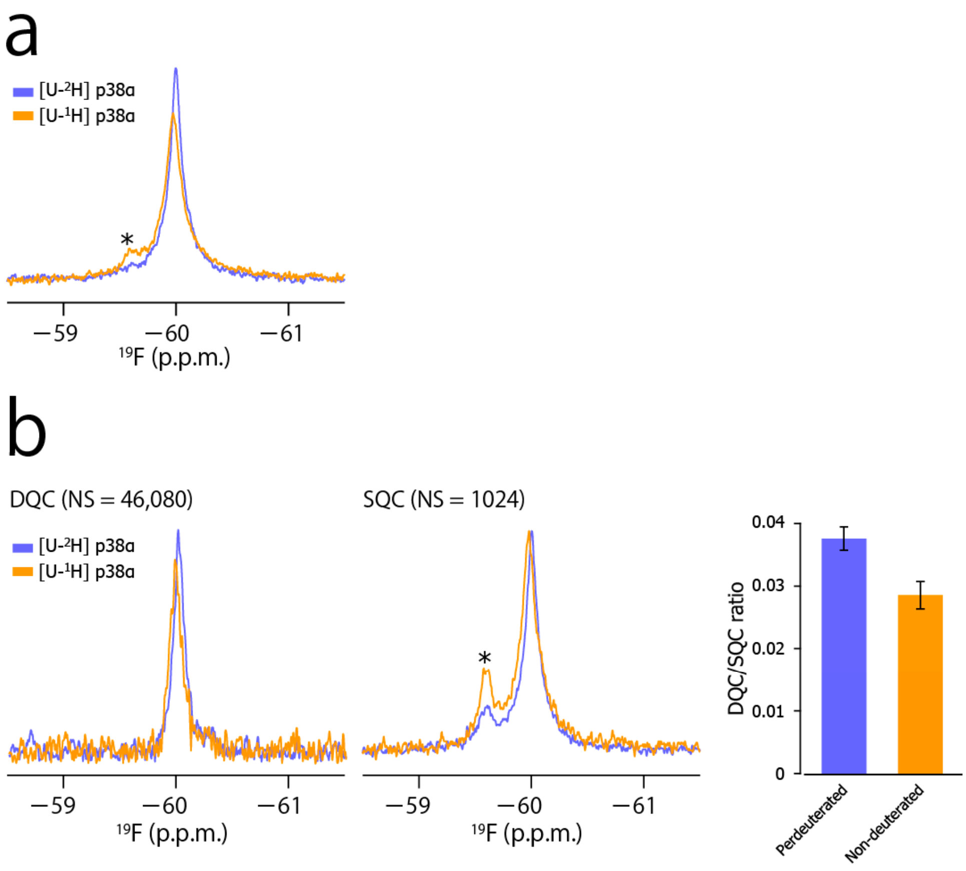

Figure S3

Supplementary Figure S3. The effect of receptor protonation on the ^{19}F linewidth and the FCT build up. (a) The ^{19}F-1D spectra of AST-487 complexed with perdeuterated (blue) and non-deuterated (orange) p38α. The weak resonance arising from the excess compound is indicated by an asterisk. (b) Comparison of the DQC (left), SQC spectra (middle), and the I_{DQC}/I_{SQC} ratio (right) of the CF_3 group of AST-487 complexed with perdeuterated (blue) and non-deuterated (orange) p38α. The numbers of transients for DQC and SQC measurements are indicated. The DQC and SQC spectra with non-deuterated p38α are scaled to make the peak heights of the SQC signals identical to those of perdeuterated p38α. Error bars are estimated from the signal-to-noise ratios.
